# Correlation of calpain sensitivity, Bradford assay instability, and electrophoretic mobility in phosphomimetic mutants of GlyT2 N-terminus

**DOI:** 10.1016/j.bbrep.2024.101734

**Published:** 2024-05-16

**Authors:** Martina Baliova, Frantisek Jursky

**Affiliations:** Laboratory of Neurobiology, Institute of Molecular Biology, Slovak Academy of Sciences, Dubravska cesta 21, 845 51, Bratislava, Slovakia

**Keywords:** Protein SDS mobility shift, Bradford assay instability, Phosphorylation, Phosphomimetic, GlyT2, Calpain

## Abstract

The glycine transporter GlyT2 plays an important role in glycine-inhibitory neurotransmission of the hindbrain and spinal cord. Its special feature is the extended N-terminus, which contains a large number of potentially phosphorylated serine and threonine residues. Due to its unstructured nature, it is difficult to address the changes introduced by potential phosphorylation. Here, we used relatively simple methods such as calpain sensitivity, Bradford instability, and SDS electrophoretic mobility shift to investigate the effect of multiple phosphomimetic mutations versus neutral mutations on GlyT2N properties. The replacement of several serines or threonines with neutral alanines did not have a significant effect on the studied GlyT2N properties. Replacement of the same residues with phosphomimetic aspartate resulted in significant alterations in calpain cleavage patterns, Bradford instability, and SDS gel protein mobility. Interestingly, a correlation between the relative intensity of the measured effects was observed, indicating that they all reflect similar structural changes introduced by potential phosphorylation *in vivo.* Results indicate that a potential single or multiple phosphorylation significantly alters the proteomic properties of the glycine transporter GlyT2 N-terminus. Assays can be helpful in the first screening of structurally significant and possibly phosphorylated residues in the N-terminus of GlyT2.

## Highlights

• Blockade of major GlyT2 N calpain cleavage sites reveals multiple calpain sensitivity.
• GlyT2 N-terminal phosphomimetic mutations cause a SDS gel mobility shift.
• GlyT2 N-terminal SDS gel mobility shift is correlated with its Bradford instability.

## 1 Introduction

Glycine transporter GlyT2 is a member of the SCL6 family of sodium-dependent neurotransmitter transporters, which regulates the glycine pool in the hindbrain and spinal cord [[1], [2], [3], [4], [5]]. The N-terminal region of GlyT2 represents an interesting, about 200 amino acid-long, cytoplasmic domain with currently unknown function [1,[6], [7], [8]]. The GlyT2N domain contains numerous phosphorylatable serines and threonines. Posttranslational modifications of amino acids in proteins, including phosphorylation, are frequently manifested by changes to their physical, chemical, and biological properties [[9], [10], [11], [12]]. Expensive and sophisticated instruments are frequently used to measure such changes; however, in some cases, the use of relatively simple laboratory techniques might also reveal very valuable information on the effect of amino acid modification on proteins. We previously discovered that the classical Coomassie Brilliant Blue G250/protein complex was unstable and showed a time-dependent wavelength absorbance shift when only unstructured N-terminal transporter protein domains were tested using Bradford reagent [8]. During our previous investigation of the calpain sensitivity of GlyT2 N-terminus we determined the position and recognition sequence of two major calpain cleavage sites [6]. In this work, we showed that when the S157D mutant was exposed to higher calpain activity, numerous low-affinity calpain cleavage sites in upstream GlyT2N region were detected. Our results presented here show that multiple neutral replacements of serines with alanine in GlyT2N did not affect the molecular weight, calpain sensitivity, or Bradford instability of the GlyT2N domain. However, when phosphomimetic mutations were introduced in identical positions, profound changes in all measured parameters were observed. The above suggests that a potential multiple phosphorylation of GlyT2N might lead to a significant change in its protein properties *in vivo.* We suggest that the structural impact of phosphomimetic or other mutations in the GlyT2 N-terminus can be quantified by the observed effects.

## 2 Material and methods

### 2.1 Plasmid construction

For the isolation of GST-rGlyT2N (1–201) protein variants containing whole rGlyT2 N-terminus, we used plasmid pGEX-5X-1-7H-T, which is the modified plasmid pGEX-5-1 (GE Healthcare, Freiburg, Germany) [13]. Shortly, at the C-terminal end of the plasmid glutathione-S-transferase (GST) coding sequence of pGEX-5-1, we inserted a linker containing seven histidines, TEV protease cleavage site, and four glycine residues between BamHI/EcoRI sites (final protein sequence insert is GST-RGIPELATMHHHHHHHELENLYFQGGGGEF-M-rGlyT2N(1–201)). Cloning of the fused protein cDNA between the EcoRI and SalI sites of the pGEX-5X-1-7H-T plasmid allowed GST fusion of rGlyT2N(1–201). GlyT2N was subsequently overexpressed and isolated using glutathione-sepharose (GE Healthcare , Freiburg, Germany) under mild conditions, as previously described [13]. Following elution of proteins with glutathione, proteins were uncoupled from the GST protein by digestion with histidine-tagged TEV protease (NEB, Ipswich, MA, USA), and the GST protein retained the histidine tag. The GST fusion partner and TEV protease was subsequently removed using nickel-charged IMAC resin (Thermo Fisher Scientific , Rockford, IL, USA), This allowed to recover a small analytical amount of rGlyT2N protein variants from the GST affinity tag, as its presence inhibits the development of the Coomassie dye absorbance dynamic [8]. The protein was ready for further analysis without tedious purification procedures and buffer exchange, which usually required a large amount of starting material. The constructs used for calpain sensitivity testing of rGlyT2N(1–154)-GST variants were prepared as follows. The PCR fragments of rGlyT2 N(1–154)-termini were prepared as follows: The internal ATG sequence in the artificially created CATATG NdeI site of the forward PCR primer matched the ATG codon of the initiating rGlyT2N(1–154) methionine. The last amino acid of GlyT2N in the C-terminal reverse primer was internal valine 154, coded by codon GTG. The last G of the valine GTG codon matched the first G of the artificially inserted GATCC BamHI recognition site. The final PCR fragment was digested with restriction nucleases NdeI and BamHI. The BglII/SalI fragment of DNA coding for glutathione S-transferase (GST) was obtained from plasmid pDS473 [14]. The flanking regions of the BglII/SalI GST fragments were as follows: N-terminus 5′-gat ctc cac cgc ggt ggc ggc cgc ttg tcc cct ata-3′ and C-terminus 5′-gat ctg gtt ccg cgt gga tcc tag tcg ac-3'. Both rGlyT2N(1–154) and GST fragments were inserted into the NdeI/SalI sites of expression vector pET21a (+) (Novagen, Merck, Darmstadt, Germany) using double ligation. The restriction sites BamHI/BglII were ligation compatible, but ligation led to the elimination of both sites in the final construct. The final constructs resulted in rGlyT2 N(1–154) terminal DNA sequences fused in frame with the N-terminal DNA coding sequence of the GST protein. The expressed protein contained rGlyT2N(1–154) with free N-terminal GlyT2 methionine, mimicking the native GlyT2N terminus. The rGlyT2N(1–154)-GST boundary was as follows: **rGlyT2N-NNNTPAVGWV**^**154**^DLHRGGGRLSPIL-GST. This truncated GlyT2N allowed us to better distinguish and count the majority of calpain cleavage sites upstream of the two calpain cleavage sites described previously [6].

### 2.2 Purification of GST fusion proteins

GST fusion proteins were isolated as previously described [13]. Resin was transferred to a 2 ml Eppendorf tube and washed several times with buffer A with EDTA reduced to 0.05 mM and Triton X-100 reduced to 0.05 %. Finally, the protein was eluted with 0.4 ml of 10 mM glutathione in 0.1 M Tris-HCl (pH 8.0).

### 2.3 Digestion with TEV protease, GST removal and Bradford instability assay

After saving a small aliquot of the eluted undigested sample, TEV protease was added directly into the eluted protein fraction and incubated overnight at room temperature. Imidazole at a final concentration of 5–10 mM was added to the samples to prevent up to 80 % loss of GlyT2N protein because of its weak interaction with nickel resin in the subsequent step. Further increase of imidazole to 20 mM increased contamination with GST protein. After saving a small aliquot of the digested sample, 0.2 ml of 50 % nickel-charged resin was added to the rest of the digested sample, and the sample was rotated at room temperature for 30 min. Following centrifugation, the supernatant was recovered for further analysis. For SDS gel, the analysed samples were mixed with 2 × SDS buffer, boiled and resolved on a 12 % polyacrylamide gel (acrylamide: bisacrylamide stock 19:1, SERVA Electrophoresis GmbH, Heidelberg, Germany). Protein bands were visualised with Coomassie G-250 staining [15].

### 2.4 Measurement of the interaction of Coomassie dye with the rGlyT2 N-terminus

The instability of the Bradford assay was measured as previously described [8]. To better visualise time-related spectral changes, the initial 1 min value was subtracted from all subsequently measured values [16]. Likely because the Bradford system is colloidal [17], we observed slight up and down variations between spectral, time-dependent increments. Because the fluctuation interval was relatively narrow, we also used averaged increment values that were independent of such fluctuations.

### 2.5 Calpain cleavage

Recombinant rat calpain was isolated as previously described [18,19] and kept at −20 °C in Tris-buffered solution containing 5 mM EDTA and 10 mM 2-mercapto ethanol (2-ME). Proteins 10 μg were diluted in 50 μl of 100 mM Tris-HCl (pH 7.4) and 2 mM CaCl_2_ and supplemented with 0.06–0.3 μg of calpain. Cleavage was performed at 25 °C and stopped by the addition of a 2X SDS buffer. Samples were resolved in 12 % PAGE.

## 3 Results

### 3.1 Multiple low-affinity rGlyT2N calpain cleavage sites are protected by phosphomimetic but not neutral mutations

**Fig. 1:**
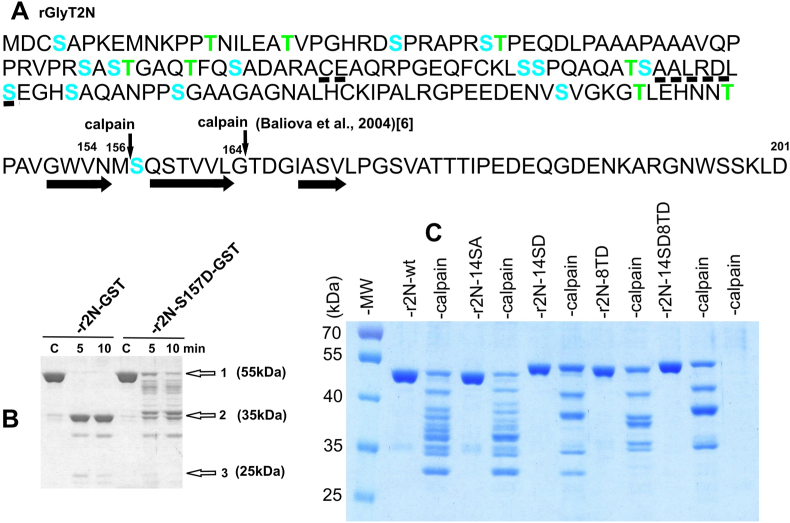
**The N-terminal region of rGlyT2 contains a number of low-affinity calpain cleavage sites.** Prediction of the GlyT2N secondary structure revealed a mostly unstructured nature. Exceptionally, the Phyre2 server (WWW.sbc.bio.ic.ac.uk/phyre2) [21] predicted helical regions (underlined by dashed lines) in positions 72–73 and 94–100 and beta-strand regions (underlined by horizontal arrows) in positions 152–171, respectively. The prediction reveals that two major calpain cleavage sites, M156/S157 and G164/T165 (A), determined previously [6], reside between the predicted beta-strand, exactly after the first amino acid following the beta-strang regions. Blockade of both sites by mutation S157D revealed the presence of numerous additional calpain sites (B). To better distinguish the spectrum of calpain cleavage sites, rGlyT2N (1–154) was fused to the GST N-terminus at the M156/S157 calpain site boundaries (C) (see material and methods for details). Mutated serines and threonines are highlighted in blue and green, respectively. Proteins (10 μg) were diluted in 50 μl of 100 mM Tris-HCl (pH 7.4) and 2 mM CaCl_2_ and supplemented with 0,06 μg (B) and 0.3 μg (C) of calpain, and cleaved at 25 °C for 5min. (For interpretation of the references to colour in this figure legend, the reader is referred to the Web version of this article.)

During our previous Western blot analyses, we ubiquitously observed the presence of a small (about 25 kDa) degradation protein fragment that was immunoreactive with anti-rGlyT2N antibodies. Although the physiological or pathological significance of such proteolysis is not clear, we previously confirmed that this fragment was created by the calcium-dependent protease calpain [6]. In subsequent work, we found that phosphomimetic S157D, but not the neutral S157A mutation, blocks both M156/S157 and G164/T165 major cleavage sites in the GlyT2 N-terminus [20]. As shown in Fig. 1A,B, 156aa, and 164aa, GlyT2N peptides should be released following a calpain cleavage at major cleavage sites [6]. They, however, cannot be distinguished on the 12 % SDS protein gel, either because of the further cleavage of the 164aa peptide or because of the similarity in molecular weight of the two fragments (Fig. 1A and B). Interestingly, according to the Phyre2 server (WWW.sbc.bio.ic.ac.uk/phyre2) prediction [21], rGlyT2N(1–201) contains three segments of beta-strand conformation in position between glycine 152 and proline 173 (Fig. 1A). The two cleavage sites are located between these segments, exactly after the first amino acid following the predicted beta-strand regions (Fig. 1A). A similar beta-strand region is also predicted by the AlphaFold model (AF-P58295-F1-model_v4.pdb) [22,23]. Fig. 1 shows that after phosphomimetic blocking of the two major cleavage sites (Fig. 1B) or when the rGlyT2N region upstream of the major calpain cleavage sites was fused with the GST at the site of valine 154 (Fig. 1C), further cleavage of the upstream region of rGlyT2N (1–154) is observed following prolonged incubation with calpain. About 5 fold more of calpain was needed for cleavage of calpain cleavage sites located in upstream GlyT2N terminus, when compared with the previously identified major cleavage calpain cleavage sites [6]. Fig. 1C shows that the phosphomimetic (S-D) but not neutral (S-A) mutations of 14 serines significantly inhibited the release of these fragments by calpain. Additional phosphomimetic mutations (T-D) of 8 threonines further reduced the number of calpain cleavage sites from about 12 to 3 (Fig. 1C). In r2N-14SD/SA proteins, the phosphomimetic but not neutral mutations also exhibited a significant SDS gel mobility shift (Fig. 1C).

### 3.2 Purification of recombinant intact and mutated rGlyT2 N-terminus free of structured proteins

**Fig. 2:**
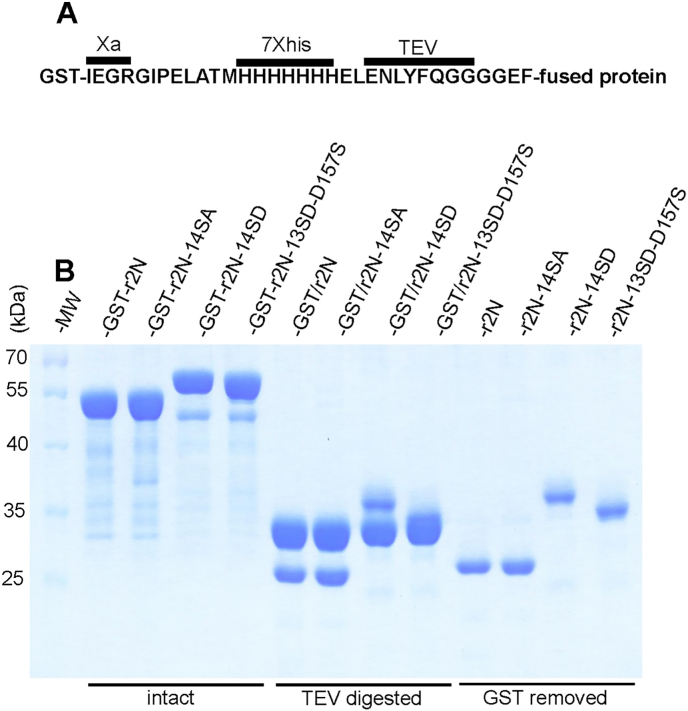
Histidine-tag-TEV-based glutathione-S-transferase (GST) fusion protein purification system. The expression cassette inserted after the GST affinity tag in the pGEX-5X-1 plasmid is indicated in panel (A). Wild-type and mutated rGlyT2N(1–201) terminal sequences were inserted after the expression cassette (GST-r2N). The proteins were overexpressed in *E. coli* and purified on glutathione sepharose (B). The left and middle underlined panels show the mobility of intact and TEV-digested phosphomimetic and neutral rGlyT2N(1–201) mutants. The right panel shows the purified rGlyT2N(1–201) proteins following the removal of the GST protein using nickel-charged IMAC resin.

We hypothesised that the potential structural changes of rGlyT2N revealed by calpain cleavage might also be reflected by previously reported dynamic interactions of GlyT2N with Coomassie dye [8]. Both the rat and human rGlyT2N termini are largely unstructured [7,8]. This feature previously allowed us to remove most of the cellular proteins by thermal denaturation and subsequent purification on ion exchange chromatography, resulting in almost homogenous rGlyT2N protein fragments. The obtained proteins were largely free of structured GST affinity tags or any additional contaminating structured proteins that could inhibit their dynamic interaction with Coomassie dye [8]. However, in potential rGlyT2N phosphorylation studies, the effect of extensive mutations of serine and threonine residues on their negatively charged phosphomimetic equivalents might radically change rGlyT2N protein properties. The thermal resistance, but mainly the isoelectric point, can change in mutated rGlyT2N to that extent, so that the binding and elution condition in the previously used ion exchange chromatography step might become unusable [7,8,16]. To avoid these obstacles, we developed a new purification approach, allowing the overexpression and purification of the native and mutated rGlyT2 N terminus applicable to virtually any other protein. Fig. 2A shows the design of the glutathione-S-transferase (GST)-based fusion protein expression cassette, allowing purification of the GST fusion proteins on glutathione sepharose under mild conditions. rGlyT2N or other proteins of interest were then disconnected from the GST fusion tag by digestion with externally supplied his-tagged TEV protease. Finally, the GST fusion tag and TEV were removed from the digested mixture by incubation with the appropriate amount of nickel-charged Sepharose resin. To prevent GlyT2N protein loss, 5–10 mM of imidazole was included into the protein samples. All of this was done in one tube using one buffer solution. The purity of the samples was checked on 12 % PAGE. We previously showed that purely disordered proteins exhibit a time-dependent spectral absorbance shift during the Bradford assay [8]. The presence of ordered proteins such as GST and BSA seems to inhibit the absorbance shift and stabilize the classical absorbance peak at 595 nm. Inhibition of the GlyT1aN16 absorbance shift by the stepwise addition of BSA [8]. Results suggest that an increase in BSA up to 0.2 mg per 0.8 mg of GlyT1aN16 does not show any inhibition. BSA, in this case, equals about 25 % of the assayed GlyT1aN16 protein. We investigated the contamination of our samples with GST using Western blot and anti-GST antibodies (Fig. 4). As an internal control, we loaded GST protein, representing about 10 % (0.1 μg) of the main loaded rGlyT2N proteins (1 μg). Results showed that the contamination of our samples is not exactly similar in all purified proteins, but it is fairly below 10 % of the main protein. We intentionally contaminated r2N-14SD sample with additional GST, representing 0.1 % of the major band. This did not significantly changed the results. Above suggests that the observed level of contamination with GST in our samples should not affect the obtained results. As shown in Fig. 2B, phosphomimetic had a significant effect on the molecular weight shift of the rGlyT2N sequence. Interestingly, single reverse mutation of calpain inhibiting the serine mutant D157S had a significant mobility effect (Fig. 2B). The effect might also be caused by various combinations of mutations, but it was beyond the scope of this work to perform such screening. However, we found that at least two mutations, S58D and S100D, had a minimal effect on the mobility shift of rGlyT2N (not shown). This confirms that not all serine phosphomimetic mutations have a significant effect on the rGlyT2N structure.

### 3.3 SDS mobility shift correlates with the calpain cleavage and Bradford assay instability of the rGlyT2 N-terminus

**Fig. 3:**
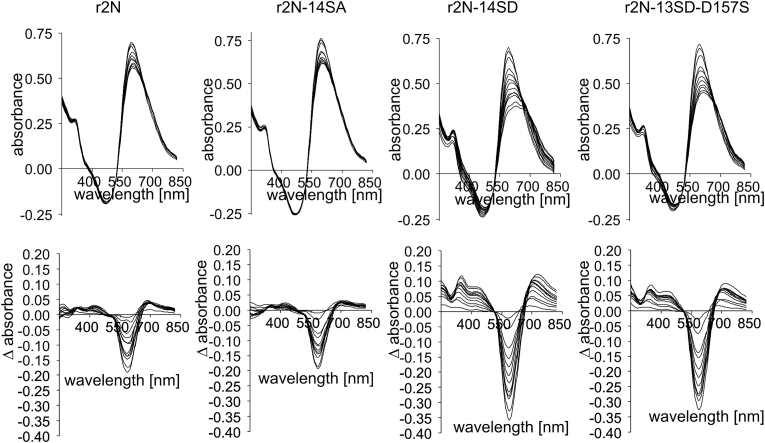
Spectral instability of the wild-type and mutated rGlyT2N(1–201), (r2N) Bradford complexes. The upper part shows time-dependent changes in the spectral absorbance profile using a 1 min time interval (A). The lower part shows differential changes in absorbance obtained by subtraction of the initial profile (1 min) from all subsequently measured profiles (B). The r2N represents the non-mutated (r2N), rGlyT2N(1–201) terminus; r2N-14SA and r2N-14SD indicate mutants where the first 14 serine residues were replaced with neutral alanine (A) and negatively charged phosphomimetic aspartic acid (D), respectively. r2N-13SD-D157S represents the r2N-14SD mutant where a single aspartic acid in position 157 was reverse mutated to original wild-type serine 157.

**Fig. 4:**
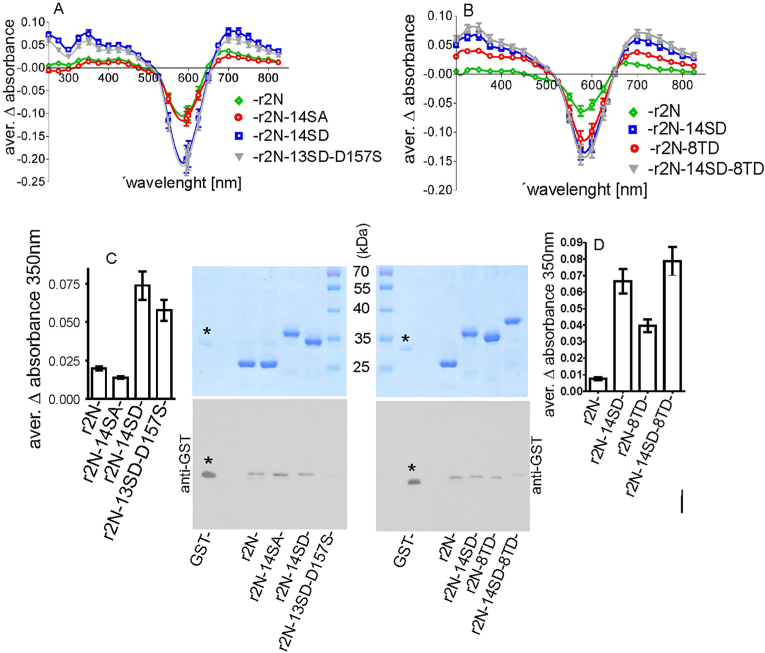
**Correlation of Bradford instability, calpain sensitivity, and SDS mobility shift in the wild type and 14SA, 14SD, and 8TD mutated rGlyT2N-termini.** The upper part of the figure (A,B) shows the averaged interval of absorbance alteration of Coomassie/protein complexes with wild-type and indicated mutants of rGlyT2N using a 1-min interval during the 10 min r2N-13SD-D157S represents the r2N-14SD mutant, where a single aspartic acid in position 157 was reverse mutated to the original wild-type serine 157. The alteration of absorbance was correlated with molecular weight shifts and was inversely proportional to the number of calpain cleavage sites (C, D). An example of the correlation of absorbance alteration at 750 nm is shown in C and D. The lower part of the figure in C and D shows the contamination of measured samples with GST, revealed by anti-GST antibody. The asterisk shows the anti-GST signal of the control GST sample, loaded in an amount representing 10 % of the major bands.

The wild-type and mutated rGlyT2N protein samples, free of structured protein contaminants, were mixed with Bradford reagent, and the time-dependent absorbance profile at 250–850 nm was measured using a 1-min interval on a Nanodrop spectrophotometer. Fig. 3 (upper panels) shows that in the case of molecular weight shift, the phosphomimetic but not neutral mutations had a significant effect on changes in Coomassie dye absorbance kinetics. The increase in differential spectral absorbance was better visualised when the initial 1-min value was subtracted from subsequent values (Fig. 3, lower panels). Fig. 3 shows 10 individual measurements using a 1-min time interval step between absorbance readings. Likely because the Bradford system is colloidal [17], we observed slight nonlinear up and down variations between spectral increments (Fig. 3, all panels). Because the fluctuation interval was relatively narrow, to further characterise absorbance instability, we used the average increment value, which was independent of such fluctuations. Fig. 4A and B shows such an averaged differential spectral absorbance increase in the range of 250–825 nm. Interestingly, this increase was generally correlated with the variation in SDS mobility shift, and it was inversely proportional to the number of calpain cleavage sites. The lower panels in Fig. 4C and D shows an example of such a correlation with the absorbance increase at 750 nm. For the relative comparison of calpain cleavage, the number of calpain cleavage sites was taken as shown in Fig. 4. To this number, two additional calpain sites were added: M156/S157 and G164/T165 [6] not present in calpain-cleaved GlyT2N proteins in Fig. 1C (see plasmid construction), but present in proteins in Fig. 2, Fig. 3, Fig. 4. The final number of calpain cleavage sites used was: r2N-wt (12), r2N-14SA(13), r2N-14SD(6), r2N-13SD-D157S(5), r2N-8TD(7), and r2N-14SD-8TD [5].

## 4 Discussion

We show here that in the case of the unstructured glycine transporter rGlyT2 N-terminus, parameters such as molecular weight shift in SDS gel, Bradford instability, and high frequency of calpain cleavage might be used to reveal the overall intrinsic structural effect introduced by phosphomimetic mutants. This is particularly interesting because rGlyT2N, containing numerous r2N14SA neutral mutants, behaves in the same way as the wild type, suggesting that r2N14SD multiple phosphomimetic mutants might resemble the potentially altered phosphorylated status of rGlyT2 *in vivo.* The calcium-dependent protease calpain is involved in neuronal remodeling and neurodegeneration, [39] and its processing is frequently regulated by phosphorylation [21,24,25]. We show here that in addition to the previously sequentially determined GlyT2N calpain cleavage sites [6], exposition of rGlyT2N to a higher concentration of calpain for an extended period of time uncovers a number of low affinity calpain cleavage sites on most of the GlyT2 N-terminus. Calpain does not have a consensus amino acid sequence, and its cleavage site is determined by a 3D structure [26]. The inhibition of some of these calpain cleavage sites by phosphomimetic mutations indicates that the structure of the local cleavage site was altered by the introduction of phosphomimetic residue. The control unspecific mutations, at least in the case of r2N14A, showed behaviour very similar to the wild type. The mechanism by which the calpain cleavage is significantly more inhibited by residues mimicking phosphorylation than neutral is presently not clear. One possibility is that calpain cleavage site association with the phosphorylation sites may be an evolutionary-selected mechanism of their control. Even though, because of the low affinity, the presence of such a large number of proteolytic fragments *in vivo* is unlikely, it cannot be completely excluded because the calpain activators have been reported previously [27,28]. While the role of released peptides may be regulatory, the creation of multiple, calpain cleavage site-specific rGlyT2 N termini could result in altered turnover, according to the N-End rule mechanism [29]. We showed here that phosphomimetic, but not neutral, mutants of potentially phosphorylatable serines and threonines increase the previously observed instability of rGlyT2N in the Bradford assay [8,16]. In contrast to neutral mutants, GlyT2N phosphomimetic mutants exhibited a SDS gel molecular weight shift. Interestingly, a correlation between the size of the shift, Bradford instability, and the number of calpain cleavage sites can be defined. Currently, we do not have an explanation for such a correlation. Since intrinsically disordered proteins are extremely heterogeneous [30,31], it remains to be determined whether this is valid only for GlyT2N or if it is a more general phenomenon. In any case, the observed effects can be used to measure the structural effect of phosphomimetic or other mutations in the GlyT2 N-terminus.

## Funding

This work was supported by the 10.13039/501100006109VEGA Grant 2/0126/21.

## CRediT authorship contribution statement

**Martina Baliova:** Investigation, Data curation. **Frantisek Jursky:** Writing – original draft, Project administration, Investigation, Funding acquisition, Data curation, Conceptualization.

## Declaration of competing interest

The authors declare no conflict of interest.
